# Supplementary material for: A comprehensive estimation of country-level basic reproduction numbers R0 for COVID-19: Regime regression can automatically estimate the end of the exponential phase in epidemic data
Source: PLoS One. 2021 Jul 13;16(7):e0254145. doi: 10.1371/journal.pone.0254145 (PMC8277067; doi:10.1371/journal.pone.0254145)
Supplement: S1 Readme — (DOCX) [file pone.0254145.s001.docx]

**README.docx**

SI_R0_Unstratified_Case_Data.zip contains 2 other files with README.docx:

1. SI_R0_Unstratified_Case_Data.docx (WORD)
2. SI_R0_Unstratified_Case_Data.xlsx (EXCEL)

The relevant Python code for R0_Unstratified_Case_Data can be found at
<https://github.com/johnlspouge/R0_Unstratified_Case_Data>.
The standalone program ARRP Version 1.1 [ARRP_1.1.zip](ftp://ftp.ncbi.nih.gov/pub/spouge/web/software/ARRP_1.1/) can be found at
<ftp://ftp.ncbi.nih.gov/pub/spouge/web/software/ARRP_1.1/>.
The ARRP Help Page is
<https://tinyurl.com/spouge-arrp>.

**(1) SI_R0_Unstratified_Case_Data.docx (WORD)**

The file contains mathematical derivations promised in the main text, along with complete URLs for the data sources in the main text.

**(2) SI_R0_Unstratified_Case_Data.xlsx (EXCEL)** has several tabs.

1. **“slope.csv”**
   **Lines 1-2** indicate “mu” and “sigma” for generation time distribution.
   The generation time is assumed to be gamma-distributed.
   **Line 3** gives the gamma-parameter kappa by formula.

   **Lines 4-164**
   1. **Columns A-I** have read a data frame from a source in Github:
      ‘Pipeline/2_ARRP/Output/slope.csv’

      **Column C**: the exponential growth rate *r* for the country
      **Column D**: the calculated error in *r***Column E**: end_date - start_date + 1
      **Column F**: the estimated start_date of the exponential epidemic phase
      **Column G**: the estimated end_date of the exponential epidemic phase
      **Column G**: the doubling time equals ln 2 / Column C
   2. **Columns J-K**
      **Column J**: the estimated r_0
      calculated from the generation-time distribution and Column C
      **Column K**: error in r_0
      calculated from the generation-time distribution and Column C-D

**Choropleths** are all computed from the table.

1. **“code2r0.csv”**
   **Lines 1-132**
   1. **Columns A-T** have read a data source in Github:
      ‘Pipeline/4_R0_ARRP/Output/code2r0.csv’

      **Column E**: the exponential growth rate *r* for the country
      **Column F**: the calculated error in *r***Column G**: end_date - start_date + 1
      **Column H**: the estimated start_date of the exponential epidemic phase
      **Column I**: the estimated end_date of the exponential epidemic phase
      **Column J**: the estimated *R*_0_
      **Column K**: the estimated error in *R*_0_
      **Column L**: the Perron-Frobenius (PF) eigenvalue of the Prem contact matrix for the country
      **Column M-T**: the PF eigenvalue of the Prem contact matrix after the indicated rows and columns are deleted [0, 1, …, *i*].

**Graphs** plot Column J (with error in Column K) against Columns L and N-R.

1. **“Fig 1”**
   A graph corresponding to Fig 1 in the main text of the article.
   It draws some data on the next 4 tabs, “ESP”, “DEU”, “AUS”, and “ARE”.
   The tabs correspond to Spain, Germany, Australia, and United Arab Emirates
   All 4 tabs have the same format as the tab “ESP”.
2. **“ESP”**
   1. **Columns A-F** have read a data source in Github:
      ‘Pipeline//2_ARRP/Output/ESP.out’
      The *.out file is an output of arrp.exe, with format explained on the
      [ARRP Help Page](https://tinyurl.com/spouge-arrp).
   2. **Columns H-K** read the output of arrp.exe
      They calculate f(y) ~ beta0 + beta1 * y for points with positive weight.
      The tab “Fig 1” plots the resulting line.
3. **“DEU”**
4. **“AUS”**
5. **“ARE”**
6. “**regression of top 5**”
   Excel regression through origin of points corresponding to the top 5 countries (DEU, TUR, USA, ESP, and IRN) for the full Prem contact matrix and the principal submatrix of the Prem contact matrix that corresponds to the population segments over age 20 years.
7. **“R0 vs gen time (mu, kappa)”**A scratch pad for calculating kappa from the estimated generation-time mean (mu) and standard deviation (std). It calculates *R*_0_ for several estimates of generation-time parameters and several values of the exponential growth rate *r*. Of the estimates displayed, the COVID-19 generation time has best estimates mu = 5.40 and std = 1.72, leading to the best estimated *R*_0_ in purple; the minimum estimated *R*_0_, in blue; the maximum estimated *R*_0_, in red. The final column displays the difference between the minimum or maximum estimated *R*_0_, and the best estimated *R*_0_. The scratch pad shows that the credible errors in *R*_0_ are not large.
