# Supplementary material for: A comprehensive estimation of country-level basic reproduction numbers R0 for COVID-19: Regime regression can automatically estimate the end of the exponential phase in epidemic data
Source: PLoS One. 2021 Jul 13;16(7):e0254145. doi: 10.1371/journal.pone.0254145 (PMC8277067; doi:10.1371/journal.pone.0254145)
Supplement: S1 Methods — (DOCX) [file pone.0254145.s002.docx]

**Justification of the Reduced Error**

**Proposition**: Let and $\sigma^{2}\left(X\right)$ be the mean and variance of $X$. Then, and $\sigma^{2}\left(\ln X\right)\approx\lambda^{-2}\sigma^{2}\left(X\right)$ under conditions discussed in the Methods.

**Heuristic**: A Taylor expansion of the function $f\left(X\right)=\ln X$ around its mean $X=\lambda$ yields

$\ln X=\ln\lambda+\frac{1}{\lambda}\left(X-\lambda\right)+...$,

because $f'\left(\lambda\right)=1/\lambda$. Drop the elided nonlinear terms. Take expectations to derive ; variances, to derive $\sigma^{2}\left(\ln X\right)=\lambda^{-2}\sigma^{2}\left(X\right)$.

**Proposition**: Let $X=n^{-1}\sum_{j=1}^{n} Y_{j}$ be a smoothed average of $\left\{Y_{j}\right\}$, where $\left\{Y_{j}\right\}$ are mutually independent Poisson variates with means $\left\{\lambda_{j}\right\}$. Let $\lambda=n^{-1}\sum_{j=1}^{n} \lambda_{j}$. Then, and $\sigma^{2}\left(X\right)=n^{-1}\lambda$.

**Proof**: Because ,

$\sigma^{2}\left(X\right)=n^{-2}\sum_{j=1}^{n} \sigma^{2}\left(Y_{j}\right)=n^{-2}\sum_{j=1}^{n} \lambda_{j}=n^{-1}\lambda$.

The approximations derived from Eq then become and $\sigma^{2}\left(\ln X\right)\approx\lambda^{-2}\sigma^{2}\left(X\right)=n^{-1}\lambda^{-1}$, so 7-day smoothing replaces $\sigma\left(\ln Y\right)\approx\lambda^{-1/2}$ with the reduced error $\sigma\left(\ln X\right)\approx n^{-1/2}\lambda^{-1/2}\approx n^{-1/2}X^{-1/2}$ (where $n=7$).

Although $\left\{Y_{j}\right\}$ are in fact mutually dependent, under many models Eq gives the dominant contribution to the variance in a smoothed average.

**Generation Times with a Gamma Distribution**

We parametrize gamma distributions with their mean $\mu$ and dispersion $\kappa$. If the generation time $T$ is gamma-distributed with parameters $\left(\mu,\kappa\right)$, its moment generating function

,

and its cumulant generating function

$K\left(s\right)=\ln M\left(s\right)=-\ln\left(1-s\mu\kappa\right)/\kappa$.

Under the $\left(\mu,\kappa\right)$-parametrization, $T$ has mean $\mu=K'\left(0\right)=\left.\mu/\left(1-s\mu\kappa\right)\right]_{s=0}$ and variance $\sigma^{2}=K''\left(0\right)=\left.\mu^{2}\kappa/\left(1-s\mu\kappa\right)^{2}\right]_{s=0}=\mu^{2}\kappa$.

As stated in Eq (1) of the main article, $R_{0}M\left(-r\right)=1$ ([Wallinga and Lipsitch, 2007](#_ENREF_2)), so

$R_{0}=\frac{1}{M\left(-r\right)}=\left(1+r\mu\kappa\right)^{1/\kappa}$.

To order $\Delta r$, the approximate error propagating from $r$ to $R_{0}$ satisfies

$\frac{\Delta R_{0}}{R_{0}}\approx\Delta\left(\ln R_{0}\right)=-\Delta\left[\ln M\left(-r\right)\right]=-\Delta K\left(-r\right)\approx K'\left(-r\right)\Delta r=\frac{\mu}{1+r\mu\kappa}\Delta r$,

so Eqs and imply Eq (2) of the main text.

**Dominant Eigenvalue of Principal Submatrices of the Prem Contact Matrix**

Recall that the dominant eigenvalue $\rho\left(\mathbf{R}\right)$ of a non-negative matrix $\mathbf{R}$ satisfies ([Kingman, 1961](#_ENREF_1))

$\rho\left(\mathbf{R}\right)=\limsup_{n\to\infty}\left[\text{tr}\left(\mathbf{R}^{n}\right)\right]^{1/n}$,

where the trace $\text{tr}\left(\mathbf{S}\right)$ of a matrix $\mathbf{S}$ is the sum $\text{tr}\left(\mathbf{S}\right)=\sum_{a=1}^{n} S_{a,a}$ of its diagonal elements. The trace is also indifferent to the order of a matrix product of square matrices $\mathbf{S}$ and $\mathbf{T}$ of the same dimensions, i.e.,

$\text{tr}\left(\mathbf{ST}\right)=\sum_{a=1}^{n} \sum_{b=1}^{n} S_{a,b}T_{b,a}=\sum_{b=1}^{n} \sum_{a=1}^{n} T_{b,a}S_{a,b}=\text{tr}\left(\mathbf{TS}\right)$.

Thus, Eqs and confirm that $\rho\left(\mathbf{ST}\right)=\rho\left(\mathbf{TS}\right)$.

Let $\mathbf{I}_{A}$ denote a matrix with element $\left(I_{A}\right)_{a,b}=1$ if $a=b\in A$, and 0 otherwise, i.e., $\mathbf{I}_{A}$ has 1s in the diagonal positions indexed by $a\in A$, and 0s everywhere else. Note $\mathbf{I}_{A}=\mathbf{I}_{A}^{2}$. As in the Materials and Methods section of the main text, $\mathbf{C}$ denotes the Prem contact matrix; $\mathbf{C}_{A}$, the principal submatrix of $\mathbf{C}$ formed by elements $C_{a,b}$ ($a,b\in A$). Then,

$\rho\left(\mathbf{I}_{A}\mathbf{C}\right)=\rho\left(\mathbf{CI}_{A}\right)=\rho\left(\mathbf{I}_{A}\mathbf{CI}_{A}\right)=\rho\left(\mathbf{C}_{A}\right)$,

with the final equality following from Eqs and , because $\mathbf{I}_{A}\mathbf{CI}_{A}$ and $\mathbf{C}_{A}$ have the same non-zero elements.

Consider a population with several subpopulations $\left(k\right)$ whose Prem contact matrices are $\mathbf{C}^{\left(k\right)}$. Consider an epidemic in the population that satisfies a modified homogeneity assumption, so that as in each expression in Eq , age-strata outside $A$ either (1) are not susceptible to disease; (2) do not transmit disease; or (3) are completely uninvolved in disease transmission. Eq confirms that in the notation of the Methods and Materials section, the basic reproduction number $R_{0}^{\left(k\right)}=\alpha C_{0,A}^{\left(k\right)}$ for each subpopulation $\left(k\right)$.

**Complete URLs for data sources**

1. [UNSD — Methodology.csv](https://unstats.un.org/unsd/methodology/m49/overview/)

<https://unstats.un.org/unsd/methodology/m49/overview/>

(downloaded 2020-12-07)

1. 3-letter [UN ISO 3166-1 alpha-3 Country Codes](https://unstats.un.org/unsd/tradekb/knowledgebase/country-code) (including Taiwan, etc.)

<https://unstats.un.org/unsd/tradekb/knowledgebase/country-code>

(accessed 2020-12-07)

1. The standalone program ARRP Version 1.1 [ARRP_1.1.zip](ftp://ftp.ncbi.nih.gov/pub/spouge/web/software/ARRP_1.1/)

<ftp://ftp.ncbi.nih.gov/pub/spouge/web/software/ARRP_1.1/>

(downloaded 2020-07-14)

1. Oxford Our World in Data (OWID) file [owid-covid-data.json](https://ourworldindata.org/coronavirus-data)

<https://ourworldindata.org/coronavirus-data>

(downloaded 2020-12-07)

1. [OWID graph](https://ourworldindata.org/coronavirus-data)

“Daily new confirmed case numbers” (smoothed over 7 days)

<https://ourworldindata.org/coronavirus-data>

(accessed 2020-12-07)

1. [Prem_2020 Contact Matrices](https://doi.org/10.1371/journal.pcbi.1005697.s002)

<https://doi.org/10.1371/journal.pcbi.1005697.s002>

(downloaded 2020-07-14)

1. <http://beautifytools.com/excel-to-json-converter.php>

Converted Excel files with Prem contact matrices to JSON.

(accessed 2020-08-08)

**Code Availability**

The code is available without restriction, under the Unlicense, at <https://github.com/johnlspouge/R0_Unstratified_Case_Data/>

## References

Kingman, J.F.C., 1961. A convexity property of positive matrices. Quart J Math Oxford 12, 283-284.

Wallinga, J., Lipsitch, M., 2007. How generation intervals shape the relationship between growth rates and reproductive numbers. Proc Biol Sci 274, 599-604.
