# Supplementary material for: A comprehensive estimation of country-level basic reproduction numbers R0 for COVID-19: Regime regression can automatically estimate the end of the exponential phase in epidemic data
Source: PLoS One. 2021 Jul 13;16(7):e0254145. doi: 10.1371/journal.pone.0254145 (PMC8277067; doi:10.1371/journal.pone.0254145)
Supplement: Attachment — Submitted filename: Reponse_to_Reviewers.docx [file pone.0254145.s004.docx]

**Response to Journal Requirements:**

2. We note that Figures 2-4 in your submission contain map images which may be copyrighted.

2b. If you are unable to obtain permission from the original copyright holder to publish these figures under the CC BY 4.0 license or if the copyright holder’s requirements are incompatible with the CC BY 4.0 license, please either i) remove the figure or ii) supply a replacement figure that complies with the CC BY 4.0 license.

Please check copyright information on all replacement figures and update the figure caption with source information. If applicable, please specify in the figure caption text when a figure is similar but not identical to the original image and is therefore for illustrative purposes only.

In accord with Note 2b, Python GeoPandas produced the world maps that replace the original Figs 2-4. The new Figs 2-4 were produced under GeoPandas open source BSD-3-Clause license.

[ <https://geopandas.org/about.html> ].

3. Any changes to the reference list should be mentioned in the rebuttal letter that accompanies your revised manuscript.

In response to Reviewer #1, I added a reference to an economic article on school re-openings in Texas, an example of the manuscript’s relevance to current COVID-19 policies.

While revising your submission, please upload your figure files to the Preflight Analysis and Conversion Engine (PACE) digital diagnostic tool, <https://pacev2.apexcovantage.com/>. PACE helps ensure that figures meet PLOS requirements. To use PACE, you must first register as a user. Registration is free. Then, login and navigate to the UPLOAD tab, where you will find detailed instructions on how to use the tool. If you encounter any issues or have any questions when using PACE, please email PLOS at [figures@plos.org](mailto:figures@plos.org). Please note that Supporting Information files do not need this step.

PACE approved the TIF files for Figs 1-6.

I thank the reviewers for their comments, which I found both thoughtful and useful.

**Reviewer #1**:

The approach and rationale are presented clearly and the mathematics are described in detail.

I thank Reviewer #1 for the encouraging comments.

The major concern with this manuscript is that the information presented is already outdated and possibly irrelevant when considering COVID-19. The primary conclusion seems to be that the younger population is not driving the spread of COVID-19 and, thus, the elderly were appropriately targeted first in the vaccine rollouts. I suggest that the authors revise the manuscript to (1) highlight their contributions to the estimation of R0 and considerations of age strata, (2) how their findings perform in the context of COVID-19, and (3) most importantly -- how these techniques/methods are relevant and may be helpful for any future pandemics. Because we are so far into the COVID-19 pandemic and subsequent vaccination strategy, using the methods described here to inform, for instance, vaccine policy recommendations does not seem currently relevant or necessary. Rather, describing how these methods fit the trends seen during the COVID-19 pandemic and translating those findings to applications in future pandemics seems much more relevant.

I have revised sentences in the abstract and discussion about vaccination. When I wrote the manuscript, vaccination strategies were still an open question. The rapidly changing realities of COVID-19 have decreased the article’s pertinence to vaccination strategies, but Googling “concern about school reopening June 2021” shows school reopening to be a current topic of concern. Accordingly, I have added a reference to [School Reopenings, Mobility, and COVID-19 Spread: Evidence from Texas](https://www.nber.org/papers/w28753) , published May 2021. My article’s findings support judicious reopening of schools.

Originally, I wrote the article as a contribution to epidemiological methods generally, as well as to COVID-19 specifically. The article’s findings still bear on policies relevant to COVID-19.

**Reviewer #2:**

The premise of this article is interesting in that it attempts to marry an analytic method & computing program from computational biology to COVID epidemic reproductive estimation.

The technical outlines and motivations are thorough, and the supplement is much appreciated.

I thank Reviewer #2 for several encouraging comments.

The paper would benefit from a limitations section.

The IMRAD format for scientific articles (Introduction, Methods, Results, and Discussion) renders a special limitations section unnecessary, because such comments clearly belong in a Discussion. Among its other revisions, therefore, the Discussion contains a new 2nd paragraph in response to this and the following comments.

First, while the novel application of regime regression to R0 estimation is notable - and the author makes a strong case for [its] benefits - there are several confounding variables impacting the results therein. Reporting and testing capacity vary significantly from country to country, and the conclusions should be squared with this reality.

One advantage of estimating an exponent as opposed to its prefactor is that the exponent is usually robust compared to the prefactor. Reporting and testing capacity, unless they vary substantially during the exponential phase, influence estimates of the prefactor much more than estimates of the exponent. The 2nd paragraph of the Discussion devotes itself to the point, while noting that preparations for an epidemic can influence the exponent through distortions of the societal steady state, and that the distortions may vary substantially between countries.

The pre-existing 3rd paragraph from the end of the Discussion also reinforces that “reporting biases” complicate interpretation.

In addition, limitations should note that the Prem contact matrices were extrapolated from 8 European countries to 152 nations. The results of R0 estimation from the Prem matrices could be a consequence of this extrapolation.

The revised 3rd paragraph from the end of the Discussion invokes the extrapolation as a possible explanation for the clusters involving from the Prem matrices. I thank Reviewer #2 for pointing out this fact.

Lines 407-409: the font appears to be slightly smaller.

Corrected, with thanks.

The work by the author is impressive, this was thought-provoking to read and review.

I appreciate the encouragement.
